# Quality of Life in HIV-Positive People in Poland Treated in the City of Bialystok: A Cross-Sectional Study

**DOI:** 10.3390/jcm12175593

**Published:** 2023-08-27

**Authors:** Marta Milewska-Buzun, Mateusz Cybulski, Anna Baranowska, Maria Kózka, Iwona Paradowska-Stankiewicz

**Affiliations:** 1 Department of Integrated Medical Care, Faculty of Health Sciences, Medical University of Bialystok, 15-096 Bialystok, Poland; mateusz.cybulski@umb.edu.pl (M.C.); anna.baranowska@umb.edu.pl (A.B.); 2 Department of Clinical Nursing, Institute of Nursing and Midwifery, Faculty of Health Sciences, Jagiellonian University Medical College, 31-501 Krakow, Poland; maria.kozka@uj.edu.pl; 3 Department of Epidemiology and Surveillance of Infectious Diseases, National Institute of Public Health NIH—National Research Institute, 00-791 Warsaw, Poland; istankiewicz@pzh.gov.pl

**Keywords:** Acceptance of Illness Scale (AIS), Human Immunodeficiency Virus (HIV), Satisfaction with Life Scale (SWLS), Short-Form Health Survey (SF-36), Quality of Life (QoL), World Health Organization Quality of Life (WHOQOL-BREF)

## Abstract

The quality of life is one of the key factors in assessing the health status of HIV-positive individuals, with its improvement considered an important goal of treatment. Assessment of the quality of life helps accurately evaluate the impact of diseases and treatment on the patient’s life. The aim of this study was to assess the quality of life of HIV-positive people in Poland using the example of patients treated in the Observation and Infection Clinic with the Subunit for HIV/AIDS Patients of the University Clinical Hospital in Bialystok, based on the evaluation of HIV-positive status acceptance in HIV patients as well as sociometric variables such as age, gender and marital status. A total of 147 patients participated in this study, including 104 men (70.7%) and 43 women (29.3%). This study was conducted between May 2019 and January 2020 in the Observation and Infection Clinic with the Subunit for HIV/AIDS Patients with the Consultation and Diagnostic Centre at the Teaching Hospital of the Medical University of Bialystok. This study used a diagnostic survey method with a modified questionnaire “Psychosocial Situation of People Living with HIV/AIDS in Poland” by Dr. Magdalena Ankiersztejn-Bartczak and the following standardised psychometric tools: the World Health Organization Quality of Life (WHOQOL-BREF), Short-Form Health Survey (SF-36), Acceptance of Illness Scale (AIS) and Satisfaction with Life Scale (SWLS). The majority of respondents (60%) reported no significant changes in their lives as a result of HIV infection. Gender was not a differentiating factor in the quality of life of people living with HIV. The variation in psychometric measures within the female and male groups was far greater than the difference between them. Marital status clearly differentiated the quality of life. The following conclusions were drawn from this study: The surveyed HIV patients presented a moderate level of quality of life, which was mainly determined by marital status. Higher quality of life was presented by married persons. Duration of infection was not correlated with quality of life. The level of acceptance of HIV infection was relatively high among respondents. A higher level of HIV acceptance was associated with a higher quality of life. The respondents presented a relatively poor level of satisfaction with life. Changing jobs, going on disability, relationship breakdown, not having a family of their own and losing friends were the key HIV-related changes in the lives of the respondents.

## 1. Background

HIV is still one of the most prevalent health problems worldwide [1,2,3]. In Poland alone, 30,092 people were diagnosed with HIV infection from the implementation of testing in 1985 until 31 December 2022 [4]. Although national governments and health organisations make investments to improve the health status of HIV patients [5,6,7,8], patients’ quality of life still calls for special attention [7].

Advances in antiretroviral therapy (ART) have improved both survival and quality of life in HIV-positive individuals. However, HIV infection and its consequences continue to have a significant impact on health-related quality of life (HRQOL), even in people who have achieved viral suppression as a result of ART [9]. Supporting HIV-positive patients in achieving HRQOL improvement requires an understanding of HRQOL determinants in this population. Studies to date have identified a number of factors that influence HRQOL among HIV-positive individuals, including the ageing process, immune status, symptoms of HIV infection, therapeutic compliance, depressive symptoms or diagnosed depression, social support and employment status [10].

The quality of life (QoL) is one of the key factors in assessing the health status of HIV patients, with its improvement considered an important goal of treatment. QoL assessment helps accurately evaluate the impact of diseases and treatment on a patient’s life [11], which means that analysing and assessing life domains that seem to be more affected by diseases is an important priority for healthcare professionals and policymakers. In the last decade or so, HRQOL research, especially in chronic diseases, has gained importance.

In Poland, there is a lack of research evidence on the determinants of QoL among people living with HIV. The existing literature on the subject has shown that gender, age, family situation, education, employment, income, viral load, TCD4+ lymphocyte count, time of diagnosis, presence of symptoms of depression and anxiety, social support, health care, use of legal and illegal drugs, adherence to antiretroviral therapy (ART), lifestyle and sexual behaviour were factors directly related to the QoL of people living with HIV [12]. Understanding QoL is essential to analysing the physical and biopsychosocial effects that HIV can cause, taking into account cultural factors specific to a given country, and thus enabling individuals to better understand themselves, their adaptation to the conditions of living with HIV and their treatment. Therefore, the aim of this study was to assess the quality of life of HIV-positive individuals in Poland using the example of patients treated in the Observation and Infection Clinic with the Subunit for HIV/AIDS Patients of the Teaching Hospital of the Medical University of Bialystok, based on the assessment of HIV-positive status acceptance and life satisfaction in HIV patient and considering sociometric variables such as age, gender and marital status.

## 2. Materials and Methods

### 2.1. Study Group

The analysis included patients living with HIV treated in the Observation and Infection Clinic with the Subunit for HIV/AIDS Patients of the Department of Infectious Diseases and Hepatology of the Teaching Hospital of the Medical University of Bialystok together with the Consultation and Diagnostic Centre of the Teaching Hospital of the Medical University of Bialystok. A total of 147 respondents took part in this study, including 104 men (70.7%) and 43 women (29.3%).

### 2.2. Study Design

This study was conducted between May 2019 and January 2020 in the Observation and Infection Clinic with the Subunit for HIV/AIDS Patients and the Consultation and Diagnostic Centre of the Teaching Hospital of the Medical University of Bialystok. The inclusion criteria were as follows: >18 years of age, confirmed HIV infection, a stay in a hospital ward or a visit to the Consultation and Diagnostic Centre and an informed and voluntary consent to participate in this study. This study was approved by the Management of the Institution and the Head of the Department. Patients’ rights, including the right to confidentiality and anonymity, were respected. In order to meet all ethical requirements during the implementation of this study, each respondent made a voluntary decision to take part in this study and could also withdraw from this study at any stage. The respondents completed the questionnaires unassisted due to the very personal nature of the questions, mainly contained in the survey; however, they were informed that if any doubts or problems with understanding the questions should arise, they could ask for clarification. Each patient hospitalised or presenting at the Diagnostic and Consultation Centre was asked to complete the questionnaires (in paper form) by themselves. Additionally, it was explained that the data obtained would only be used for research purposes. Patients completed the questionnaires in the patient room (in-patients) at the Consultation and Diagnostic Centre or at home, handing them in during the next visit. All questionnaires were returned in a sealed return envelope. This study was conducted with the involvement of persons in close contact with the people living with HIV, i.e., infectious disease doctors and nurses, and during direct meetings with HIV patients. A total of 198 questionnaires were distributed, of which 159 (80.30%) were returned, including 12 incomplete questionnaires (19.08%), which were discarded during the analysis. A total of 147 questionnaires were included in the analysis—a response rate of 74.25%.

### 2.3. Measures

This study used a diagnostic survey with the use of a modified questionnaire “Psychosocial situation of people living with HIV/AIDS in Poland” by Dr. Magdalena Ankiersztejn-Bartczak, President of the Social Education Foundation in Warsaw. Written consent of the author was obtained for the use of the questionnaire. Additionally, the following standardised psychometric scales were used in this study: the World Health Organization Quality of Life (WHOQOL-BREF), Short-Form Health Survey (SF-36), Acceptance of Illness Scale (AIS) and Satisfaction with Life Scale (SWLS).

#### 2.3.1. Modified Version of “Psychosocial Situation of People Living with HIV/AIDS in Poland” [13]

The survey questionnaire consists of 59 questions. All questions require a specific choice of one or more answers. Some questions are additionally open-ended, giving the respondent the opportunity to address the question more broadly or to voice his/her own view/suggestion. The questions are structured in a way that is clear and comprehensible for the patient and refer to a retrospective analysis of the situation since receiving the diagnosis and an assessment of various aspects of life, including those relating to the last twelve months only. The questions in the questionnaire are grouped into four thematic categories:

1. Sociodemographic characteristics, including age, education, place of residence, income and housing conditions.
2. Diagnosis and confirmation of HIV infection.
3. The impact of the diagnosis on life.
4. Public reactions to information about infection.

#### 2.3.2. The World Health Organization Quality of Life (WHOQOL-BREF)

The WHOQOL-BREF questionnaire contains 26 questions and is used to measure quality of life in four domains: psychological health, physical health, environment and social relationships [14]. The psychological domain includes positive and negative feelings, physical appearance, religion and spirituality, self-esteem, faith, sense of concentration, thinking, memory and learning. The physical domain includes rest and sleep, discomfort and pain, mobility, daily activities, dependence on medication and treatment and ability to undertake work. In the environmental domain, respondents assessed their financial resources, sense of security, freedom, access to and quality of health care, relationships with the immediate environment, housing conditions, opportunities for rest and recreation, opportunities for acquiring new information and skills and transport. The social domain includes interpersonal relationships, satisfaction with sexual life and social support [14]. Additionally, the WHOQOL-BREF contains two questions that are analysed separately. Question 1 asks about the individual’s overall perception of their quality of life, and question 2 asks about the individual’s overall perception of their health. Responses are scored on a 5-point scale (low score of 1 to high score of 5), with a reverse interpretation in three questions, i.e., 5 is the lowest value and 1 is the highest value. A maximum score of 20 can be obtained in each of the domains indicated above. The higher the score, the better the patients’ quality of life [14]. Cronbach’s alpha coefficient values for each of the six domains range from 0.71 (for the social domain) to 0.86 (for the environmental domain). The overall Cronbach’s alpha coefficient for the scale is 0.84 [14].

#### 2.3.3. Short-Form Health Survey (SF-36)

The SF-36 Quality of Life Assessment Questionnaire was created in 1988 and is one of the most widely used generic tools for measuring health-related quality of life. It is designed for subjective assessment of health status [15]. Due to its high diagnostic sensitivity, it can be used even in the early stages of disease. The tool consists of 36 questions in 11 categories to distinguish eight aspects of quality of life, such as the following [15]:

- Physical function—range of typical physical daily activities (10 items);
- Role limitations due to physical problems—the effect of physical health on daily activities (4 items);
- Bodily pain—severity of physical pain and its impact on daily activities (2 items);
- General health perceptions—i.e., the patient’s self-reported overall health in relation to their expectations and perception of health (5 items);
- Vitality—level of vital energy and fatigue (4 items);
- Social functioning—impact of health on social functioning (2 items);
- Role limitations due to emotional problems—impact of emotional problems on daily functioning (3 items);
- Perceived mental health—quantitatively classified as nervousness, irritability, depression and happiness (5 items).

Additionally, health status is assessed in comparison with the health status one year earlier. The type of answers to individual questions varies from dichotomous (yes/no) to 3-, 5- and 6-point Likert scale responses. Respondents’ answers are normalised so that the resulting QoL measures range from 0–100, with 0 always indicating the worst QoL and a score of 100 indicating the best QoL. Cronbach’s alpha coefficient values range from 0.73 (social functioning) to 0.96 (role limitations due to physical health; role limitations due to emotional problems; and vitality) [15].

#### 2.3.4. Acceptance of Illness Scale (AIS)

The AIS questions address specific difficulties and limitations arising from one’s health status. The AIS can be used to measure acceptance of any illness. It contains eight statements describing negative health consequences in the form of limitations due to the illness, lack of self-sufficiency, the sense of being dependent on others and reduced self-esteem [16]. In each statement, the respondents identify their current health status on a five-point Likert scale with the following responses: 1—strongly agree, 2—agree, 3—not sure, 4—disagree and 5—strongly disagree. Strongly agree indicates poor adaptation to the disease, while disagree indicates disease acceptance. The overall score ranges from 8 to 40. The degree of acceptance is defined by three score ranges. A score of 8 to 18 indicates a lack of illness acceptance, 19 to 29 represents an average level of acceptance and 30 to 40 defines a high level of acceptance of the health situation. The reliability of the Polish version of the AIS is similar to that of the original version, with a Cronbach’s alpha coefficient of 0.82 [16].

#### 2.3.5. Satisfaction with Life Scale (SWLS)

The Satisfaction with Life Scale (SWLS) consists of five statements, which are rated by the respondent on a 7-point scale by selecting one of the possible answers [17]. The respondent assesses to what extent each of the statements applies to his or her life to date, with the following responses: 1—strongly disagree, 2—disagree, 3—slightly disagree, 4—neither agree nor disagree, 5—slightly agree, 6—agree and 7—strongly agree. The answers are scored, and the total score represents the overall degree of satisfaction with life. The scores range from 5 to 35, and the higher the score, the greater the sense of satisfaction with life. Sten scale is used for interpretation, where scores in the range of 1–4 stens (a score of 5–17) represent low values, 5–6 stens (a score of 18–23) represent average values and 7–10 stens (a score of 24–35) represent high values. A score of 20 represents a neutral point on the scale and means that the respondent is neither satisfied nor dissatisfied to any degree. A score of 5–9 indicates extreme dissatisfaction with life, while a score of more than 30 indicates high satisfaction with life. The Cronbach’s alpha coefficient is 0.87 [17].

### 2.4. Procedure and Ethical Considerations

This study was conducted following the recommendations of and was reviewed and approved by the Bioethics Committee of the Medical University in Bialystok (statute no. R-I-002/237/2019). All participants gave written informed consent in accordance with the Declaration of Helsinki.

### 2.5. Statistical Analysis

Statistica 13.3 (StatSoft Polska, Krakow) was used for statistical analysis.

Descriptive statistics and statistical inference, with the choice of methods determined by the type and distribution of the characteristics analysed, were used for statistical analysis.

The descriptive section presents the numerical and percentage distribution of nominal characteristics, while for measurable characteristics (mainly psychometric measures), selected descriptive statistics were determined: arithmetic mean ($\bar{x}$), median (middle value) (Me), maximum value (max.) and minimum value (min.), standard deviation (SD) and lower and upper quartile (*c*_25_ and *c*_75_).

The relationships between the different aspects of QoL were also assessed. For this purpose, Spearman’s rank correlation coefficients between the different measures were determined.

Various data analysis techniques were used to assess the variation in the quality of life of respondents, depending on their demographic profile and other factors. Spearman’s rank correlation coefficient was used for any variables whose values can be ordered in ascending order, such as age or time since diagnosis.

When psychometric measures were compared between groups, i.e., when the independent factor was nominal (e.g., for gender, marital status, etc.), descriptive statistics were determined in the compared groups and the significance of the differences between them was assessed using the Mann–Whitney test for two groups or the Kruskal–Wallis test for more groups.

Multiple regression analysis was used in this study. Individual psychometric scales were adopted as dependent variables, and marital status was taken into account as an independent variable and adjusted for gender, age and time since HIV diagnosis. The conducted analysis did not show the presence of confounding factors; therefore, it was not included in the further part of this article.

The results of all the above-mentioned statistical tests were interpreted using the probability (*p*) value, assuming a statistically significant relationship at *p* < 0.05.

## 3. Results

The mean age of the respondents ($\bar{x}$) was 42.5 years with a standard deviation (SD) of 10.4 years. The youngest respondent was 22 years old, while the oldest respondent was 77 years old. Respondents with secondary education (36.1%) were the predominant group. Urban residents, with a predominance of those living in cities with more than 200,000 inhabitants (residents of Bialystok), dominated in the study group. Rural respondents accounted for less than 14%. Almost one in two patients was single. Married respondents accounted for almost 30% of the surveyed group. Detailed data are shown in Table 1. The mean duration of diagnosis was almost 12 years (11.8 ± 7.7), with less than 10 years since diagnosis in half of the study group.

The majority of respondents (60%) reported no significant changes in their lives as a result of HIV infection. Some respondents (16%) received a disability pension as a result of the infection, thus giving up their employment. For some respondents (28%), HIV infection meant the breakup of their family or the loss of friends. About 11% of respondents consciously gave up starting their own family. Only 2% of those infected started substance abuse therapy after learning about their infectious status (Table 2).

Almost one in five respondents (18%) changed their job after learning about their HIV status. Physical inability to perform current job (work that was too physically demanding) was reported as the main reason for this change.

The majority of patients surveyed positively rated their personal social relationships with other people—47% were satisfied and 11% were very satisfied. Only 7% and 3% of respondents were dissatisfied and very dissatisfied in this respect, respectively. Moderate satisfaction was reported by 31% of respondents. When assessing personal relationships with one’s family after receiving the HIV diagnosis, it was found that about ¾ of respondents did not feel that they had deteriorated (72.1%). For 40 respondents (27.2%), the diagnosis had a negative impact on family relationships. One person did not answer the question.

The level of infection acceptance was rather high in the study population. The median AIS score was 31. The mean satisfaction with life score in the study group was about 18, which is closer to the lower limit of possible values and thus indicates that the satisfaction with life in the study group was rather poor. The quality of life assessed with the SF-36 questionnaire varied depending on the aspect of life in question. Physical functioning was rated high (median up to 95), while the rating of vitality was very low. The mean for the somatic domain of the WHOQOL-BREF questionnaire was 14.5, while the mental domain score was lower (12.5). Detailed data are shown in Table 3.

Measures of QoL and life satisfaction (SWLS, SF-36 and WHOQOL-BREF) were strongly correlated with each other, with the weakest correlations at about 0.50 and with the two main SF-36 measures found to be the most strongly correlated (correlation coefficient above 0.90). Measures from the different questionnaires were also strongly correlated with each other, e.g., the SF-36 measures with the WHOQOL-BREF measures at a level of no less than 0.70. The SWLS measure was slightly less correlated with the other variables. Detailed correlation coefficients are shown in Table 4.

The correlation coefficients for infection acceptance and QoL measures were positive, which means that the fact of infection acceptance had a positive impact on the participants’ quality of life (Table 5).

The analyses showed that gender was not a differentiating factor in the quality of life of people living with HIV. The variation of psychometric measures within the group of women and men was definitely greater than the difference between them, which was evident when comparing standard deviations against the difference of means for both groups (*p* < 0.05).

No significant differences were found for any of the SF-36, SWLS and AIS measures. The only result close to being statistically significant (test probability values of *p* minimally above 0.05) was found for the WHOQOL-BREF measures. It was found that men’s quality of life in the environmental domain was minimally higher. However, this was a difference of <1 (mean score was 13.9 for women and 14.5 for men), so the substantive value of these results appeared to be small (Table 6).

Another analysis looked at the relationship between age and time since diagnosis and the quality of life in people living with HIV. It was observed that time since diagnosis was not associated with QoL, while in the case of age, there were two statistically significant correlations, but both were of very low strength (*r*_S_ < 0.30). The negative sign of the correlation coefficient indicated that the QoL in physical functioning, total physical domain and total quality of life lightly declined with age. No statistically significant correlations were found between age and time since diagnosis and QoL measures in the WHOQOL-BREF questionnaire. Also, none of the other measures considered (SWLS and AIS) showed a relationship with age or time since diagnosis (Table 7).

In contrast to gender, age and time since diagnosis, marital status clearly differentiated the quality of life. Married people showed by far the most favourable measures in the SF-36. Their overall quality of life was about 10 points higher than in the other two groups. Marital status was a similar differentiating factor in the quality of life measured with the WHOQOL-BREF. The least pronounced differences were in the social domain (*p* = 0.0302), with *p* ≤ 0.001 for the other three measures. In contrast, marital status did not differentiate the level of infection acceptance. For life satisfaction, as in the case of QoL measures, the differences between groups were highly statistically significant. A comparison of mean values indicated that married respondents showed the highest life satisfaction (Table 8).

## 4. Discussion

HIV infection can significantly compromise the quality of life of the affected person. This impact can be complex and involve various aspects of life, with a number of demographic, social, economic and emotional interactions, as well as factors related solely to the infection, such as its duration, comorbidities, health limitations or adverse effects of antiretroviral therapy. The introduction of effective treatment for HIV infection has not only extended the lifespan of these patients but also allowed for a change in the perception of HIV infection by both the patients themselves and those around them. HIV infection itself is now considered a chronic condition rather than a death sentence for the patient. With this in mind, the holistic approach has become an extremely important part of the management strategy for these patients. In addition to the purely medical aspects, it includes an assessment of the patient’s quality of life.

Our study showed that, in contrast to the respondents’ age, duration of infection was not correlated in any way with the quality of life. However, QoL in terms of physical function slightly decreased with age (*p* = 0.003), but the strength of these correlations was low. This may be due to the fact that the mean age of respondents was 42 years (35% of the study group), followed by 30–39 years (34% of the study group). A cross-sectional study by Kitilya et al. [18] in a group of 272 people living with HIV in Tanzania, who were similar in terms of age to our study group, found that these individuals were also characterised by poorer QoL in terms of physical function, which translated into reduced activity and physical capacity [18]. Emlet et al. [19] assessed quality of life in more than 200 homosexual and bisexual men with HIV in the US. They found that neither age nor duration of infection were related to QoL in terms of physical health. However, these results decreased with an increase in the number of comorbidities and the resulting activity limitations. Interestingly, experiencing violence resulting from the stigma of HIV-positive status was a significant factor decreasing physical functioning. Additionally, these results positively correlated with a sense of being in control over one’s life [19]. In conclusion, the results of studies on the relationship between illness duration and the age of patients and the quality of life in the physical function domain are inconclusive, with some researchers showing a deterioration of QoL in the somatic aspect with age and other studies showing no such relationship. Wellbeing, in terms of physical function, is a very important element that enhances the quality of life in all patients.

The prevalence of pain in people living with HIV ranges from 24% to 93% [20]. There are most often pain syndromes due to HIV infection, secondary immunosuppression and diagnostic and therapeutic management, and their frequency increases with the progress of the disease, significantly limiting the patients’ functioning and preventing them from fulfilling social and occupational roles [20]. In our study, pain measured with the SF-36 questionnaire was not a factor significantly affecting the quality of life of respondents. Nevertheless, almost one in five respondents had changed jobs due to HIV infection. They reported physical inability to perform their previous job (work that was too physically demanding) as the main reason for this change. The above analyses showed that physical activity among HIV/AIDS patients is related to a number of complex factors that should be considered in the treatment process. However, for the impact of physical activity on the quality of life to be positive, it is important to remember that moderate-intensity exercise improves immune function in people with HIV/AIDS, while high-intensity exercise has an immunosuppressive effect [21]. HIV-positive individuals should receive professional counselling in this regard.

Since studies on gender differences in relation to health-related quality of life in HIV/AIDS patients are scarce and contradictory, we decided to analyse this factor in our study. SF-36, SWLS and AIS measures showed no statistically significant differences. It was only in the case of measures calculated from the WHOQOL-BREF that one result close to statistical significance was found (*p*-value minimally above 0.05). It was found that men’s quality of life was minimally higher in the environmental domain. However, this difference accounted for less than one point. A similar analysis was conducted by Rzeszutek [22] in a study aimed at identifying gender differences in health-related quality of life and coping strategies in people living with HIV. The study involved 444 men and 86 women with HIV. It was a cross-sectional study in which health-related QoL was also assessed using the WHOQOL-BREF questionnaire. There were no statistically significant differences in individual QoL domains that could be directly attributed to gender. However, statistically significant interactions were found between participants’ gender and coping strategies in relation to the domains of health-related QoL. It was found that access to emotional support had a positive impact on the quality of life in the environmental, social and psychological domains only in the female group, while in the male group, the use of emotional support was negatively correlated with the somatic domain [21]. A study by Gebremichael et al. [23] on the impact of gender on health-related QoL in people living with HIV/AIDS in Ethiopia suggests that QoL differs significantly between genders. A comparative cross-sectional study was conducted among 520 patients with HIV/AIDS receiving antiretroviral therapy using the WHOQOL-HIV BREF questionnaire. It found that women had significantly lower QoL in the somatic, psychological, social and environmental domains compared to men, with the exception of social and spiritual relationships [23]. In their meta-analysis of 49 studies conducted in highly developed countries, Degroote et al. [12] concluded that gender was sometimes a differentiating factor between men and women with HIV in terms of quality of life, but some of the results did not confirm this relationship. In those cases where such a difference did occur, women scored lower than men [12]. The impact of gender on QoL of people living with HIV was also investigated by Fumaz et al. [24] among an HIV-positive Spanish population. The sample consisted of 744 patients, of whom 538 (72.3%) were men. Analysis of the results showed that women exhibited more emotional distress and negative moods related to HIV infection, were less satisfied with their body image and experienced more discriminatory behaviour than men [24]. Thus, as can be seen, the relationship between gender and QoL in seropositive individuals cannot be stated unequivocally, and those that do occur may be due to, for example, the sociocultural background of the community. As quality of life is influenced by multiple independent factors, it may be that the observed gender differences are secondary to these factors.

In our study, single people made up 40% of the study group, and therefore, we assessed whether the respondents’ marital status had an impact on their quality of life. The results clearly confirmed this relationship, in favour of those married. The highest level of QoL in this group of respondents was found in the context of the SF-36 measures, where the overall QoL of the respondents was found to be on average 10 points higher than in the other two groups. In a similar way, marital status differentiated QoL measured with the WHOQOL-BREF questionnaire. The least pronounced differences were found for the social domain (*p* = 0.0302), with probability values *p* ≤ 0.001 for the other three measures. The marital status of respondents did not, however, differentiate the level of infection acceptance (AIS). For satisfaction with life (SWLS), as for the QoL measures, the differences between the groups proved highly statistically significant (*p* = 0.0007). A comparison of the mean or median showed that married persons were characterised by the highest life satisfaction. The positive impact of the marital relationship on the QoL in seropositive individuals has been confirmed by numerous studies. Joulaei et al. [25] found a statistically significant association between marital status and quality of life, with married patients presenting a significantly higher QoL compared to single patients. Khademi et al. [1], who conducted their study in a sample of 364 HIV-positive patients, also confirmed that being married vs. single correlated positively with overall QoL, as well as psychological and social relationships in people living with HIV (*p* ˂ 0.001) [1]. There are a number of studies [26,27,28] that clearly show a strong relationship between marital status and QoL among seropositive people, demonstrating that the absence of a formal marriage was significantly associated with lower QoL among HIV-positive people. In conclusion, the impact of the marital relationship on the QoL of HIV-positive people is definitely positive and confirmed. The problem, on the other hand, may be related to those relationships in which there is serodiscordance, i.e., only one partner is seropositive. Determining the level of QoL and marital satisfaction in HIV seroconcordant and HIV serodiscordant couples, compared to healthy couples, became the focus of a study by Faraji et al. [29]. Quality of life and marital satisfaction were assessed using a short version of the World Health Organization Quality of Life Questionnaire (WHOQOL-BREF) and the ENRICH questionnaire, respectively. The study confirmed that quality of life and marital satisfaction were significantly higher in healthy couples than in the two other study groups (*p* < 0.001). In contrast, there was no significant difference between QoL and marital satisfaction between seroconcordant and serodiscordant groups; however, marital satisfaction was higher in men than in women in these groups [29].

### Limitations

Research on the quality of life in HIV-positive persons is a complex process that requires specialised knowledge, interpersonal skills and, above all, the maintenance of a high ethical level at every stage of the study. In order to make the above assessment as reliable as possible, it becomes necessary to study many aspects of life, including the intimate sphere, which is often an extremely difficult subject for HIV-positive patients. The assistance in completing the survey and questionnaires offered to patients was not met with respondents’ approval. It should be emphasised that in order to maintain a high ethical standard, respondents were informed that they could withdraw from this study at any stage.

In analysing the research data, it was found that one or more questions, mainly related to the intimate sphere, were not answered. Given the very broad scope of this study, and therefore the necessity to complete an extensive survey and four questionnaires (SF-36, AIS, WHOQOL-BREF and SWLS), it can be assumed that some respondents may have consciously skipped selected questions. It can also be assumed that this may have resulted from accidental or deliberate omission, due to the overly intimate nature of the questions or the voluminous nature of the questionnaires. The questionnaires completed by the respondents were handed over to the medical staff (doctor, nurse) involved in the patient’s therapy. Although each questionnaire was prepared in such a way that those receiving it from the patients did not have direct access to the contents (a sealed return envelope was used), it can be assumed that some respondents may have had concerns about whether the confidentiality principle applicable to the survey would actually be maintained. In the future, consideration should be given to providing a specially prepared and secured box for this purpose. It is also possible that some questions were incomprehensible to respondents. The place where the survey was completed may also have been the reason why some questions were left unanswered. Some respondents took the questionnaire home, while others did not for personal reasons. This group chose to complete the questionnaire either while at the doctor’s appointment (in a specially prepared room) or in the patient room (the group being hospitalised). This could have been a reason for respondents to be distracted or to fill in the forms superficially, without deeper reflection, due to haste or impatience (having to return to work, home duties or public transport schedules). Also, some of those who decided to take the questionnaire home did not return them. It must be assumed that they simply did not intend to take part in the survey. Therefore, the quality of life of patients treated in the Observation and Infection Clinic with the Subunit for HIV/AIDS Patients at the Teaching Hospital of the Medical University of Bialystok does not refer to the total group of seropositive individuals attending the Centre. In the future, it would be advisable to consider involving interviewers who would provide assistance in completing the questionnaires or using fewer survey tools.

Another limitation was small sample size, which may result in difficulty in achieving statistical significance, particularly for groups with small size (females, etc.). In addition, a survey questionnaire that was too extensive (several similar scales) could cause fatigue of respondents with the survey, in particular with similar content of questions regarding QoL.

## 5. Conclusions

The quality of life in people living with HIV treated at the Observation and Infection Clinic with the Subunit for HIV/AIDS Patients at the Teaching Hospital of the Medical University of Bialystok was moderate.

The quality of life of people living with HIV was mainly determined by marital status. Higher quality of life was presented by married persons. Duration of infection was not correlated with the quality of life.

The level of acceptance of HIV infection among respondents was relatively high. A higher level of acceptance was associated with better quality of life. Respondents presented relatively low levels of life satisfaction.

Some of the most important changes in respondents’ lives as a result of HIV infection included changing jobs, going on disability, relationship breakdown, not having a family of their own and losing friends.

## Figures and Tables

**Table 1:** Sociodemographic characteristics of respondents (n = 147).

Sociometric Variable		n	%
Gender	female	43	29.3%
	male	104	70.7%
Age (years)	<30	14	9.5%
	30–39	50	34.0%
	40–49	51	34.7%
	50–59	22	15.0%
	60–69	8	5.4%
	≥70	2	1.4%
Education	primary	31	21.1%
	vocational	37	25.2%
	secondary	53	36.1%
	higher	26	17.7%
Place of residence	rural	20	13.6%
	city up to 50,000	29	19.7%
	city of 50–100,000	39	26.5%
	city of 100–200,000	13	8.8%
	city > 200,000	46	31.3%
Marital status	single	71	48.3%
	married	42	28.6%
	divorced	28	19.0%
	widow/widower	6	4.1%

**Table 2:** Impact of HIV infection on changes in respondents’ life plans (n = 147).

Life Plan Changes Related to the Detected Infection	n	% ^1)^
no change	89	60.5%
disability pension	23	15.6%
relationship breakdown	20	13.6%
no family of one’s own	16	10.9%
loss of friends	11	7.5%
loss of contact with the family	9	6.1%
moving to another city	6	4.1%
change in friends	6	4.1%
loss of job	4	2.7%
start of substance abuse treatment	3	2.0%
lack of choice	1	0.7%

^1)^ The sum does not have to equal 100% as any number of response options could be indicated.

**Table 3:** Descriptive statistics of the scales used in this study (n = 147).

Psychometric Measures		$\bar{x}$	Me	SD	*c*_25_	*c*_75_	Min.	Max.
AIS		28.2	31	9.5	20	36	8	40
SWLS		17.9	20	8.2	11	23	5	35
SF-36	Physical function	79.3	95.0	27.3	65.0	100.0	0.0	100.0
	Role limitations due to physical problems	59.9	75.0	43.4	0.0	100.0	0.0	100.0
	Pain	65.5	77.5	30.5	37.5	90.0	0.0	100.0
	General health	46.9	50.0	20.4	37.5	62.5	0.0	87.5
	Physical domain	69.8	78.6	26.0	51.7	90.6	0.0	98.6
	Role limitations due to emotional problems	70.3	100.0	42.9	33.3	100.0	0.0	100.0
	Vitality	42.8	43.8	14.3	31.3	50.0	6.3	75.0
	Social functions	74.2	75.0	25.3	50.0	100.0	12.5	100.0
	Wellbeing	54.6	55.6	21.1	42.2	68.9	0.0	100.0
	Mental domain	56.9	60.6	18.3	43.3	70.0	8.1	88.3
	Total quality of life	63.3	69.9	20.7	50.6	78.9	5.6	91.4
WHOQOL-BREF	Somatic domain	14.5	14.9	3.1	12.6	16.6	5.7	20.0
	Psychological domain	12.5	12.7	3.3	10.0	15.3	4.7	18.0
	Social domain	12.7	13.3	4.0	9.3	16.0	4.0	20.0
	Environment	14.3	15.0	2.5	13.5	16.0	5.5	19.0

Abbreviations: AIS—Acceptance of Illness Scale, SF-36—Quality of Life Assessment Questionnaire, SWLS—Satisfaction with Life Scale, WHOQOL-BREF—World Health Organization Abbreviated Quality of Life Questionnaire, $\bar{x}$—arithmetic mean, Me—median, SD—standard deviation, *c*_25_—lower quartile, *c*_75_—upper quartile, Min.—minimum, Max.—maximum.

**Table 4:** Correlation coefficients for SWLS, SF-36 and WHOQOL-BREF measures (n = 147).

Quality of Life Measures	(1)	(2)	(3)	(4)	(5)	(6)	(7)	(8)
(1) SWLS	1	0.45	0.54	0.51	0.61	0.54	0.52	0.50
(2) Physical domain (SF-36)	0.45	1	0.68	0.91	0.74	0.70	0.39	0.47
(3) Mental domain (SF-36)	0.54	0.68	1	0.90	0.63	0.68	0.52	0.47
(4) Total quality of life (SF-36)	0.51	0.91	0.90	1	0.74	0.73	0.47	0.50
(5) Somatic domain (WHOQOL-BREF)	0.61	0.74	0.63	0.74	1	0.77	0.49	0.65
(6) Psychological domain (WHOQOL-BREF)	0.54	0.70	0.68	0.73	0.77	1	0.58	0.64
(7) Social domain (WHOQOL-BREF)	0.52	0.39	0.52	0.47	0.49	0.58	1	0.48
(8) Environment (WHOQOL-BREF)	0.50	0.47	0.47	0.50	0.65	0.64	0.48	1

Abbreviations: SWLS—Satisfaction with Life Scale, SF-36—Quality of Life Assessment Questionnaire, WHOQOL-BREF—World Health Organization Abbreviated Quality of Life Questionnaire. All correlations were statistically significant at *p* < 0.05 (Spearman’s rank correlation coefficient).

**Table 5:** Correlations between the acceptance of infection and the SWLS and WHOQOL-BREF measures (n = 147).

Psychometric Measures	Infection Acceptance (AIS)
Life satisfaction (SWLS)	0.53
Somatic domain (WHOQOL-BREF)	0.49
Psychological domain (WHOQOL-BREF)	0.52
Social domain (WHOQOL-BREF)	0.50
Environment (WHOQOL-BREF)	0.42

Abbreviations: SWLS—Satisfaction with Life Scale, WHQOL-BREF—World Health Organization Abbreviated Quality of Life Questionnaire, AIS—Acceptance of Illness Scale, *p*—probability value. All correlations were statistically significant at *p* ≤ 0.001 (Spearman’s rank correlation coefficient).

**Table 6:** Relationship between gender and quality of life as measured with SF-36, WHOQOL-BREF, AIS and SWLS (n = 147).

Psychometric Measures		Gender				*p*
		Women (n = 43)		Men (n = 104)		
		$\bar{x}$	SD	$\bar{x}$	SD	
Quality of life (SF-36)	Physical function	76.4	27.4	80.4	27.4	0.2593
	Role limitations due to physical health problems	59.9	46.7	59.9	42.2	0.8904
	Pain	60.5	35.0	67.6	28.4	0.3404
	General health	49.4	20.6	45.8	20.4	0.4512
	Role limitations due to emotional problems	75.2	41.2	68.3	43.7	0.4589
	Vitality	43.6	14.7	42.5	14.2	0.4667
	Social functions	70.3	26.6	75.8	24.7	0.3030
	Wellbeing	55.7	22.4	54.2	20.7	0.5711
	Physical area	68.0	27.6	70.6	25.5	0.7073
	Mental domain	57.9	19.7	56.4	17.7	0.5039
	Total quality of life	62.9	22.0	63.5	20.2	0.9408
WHOQOL-BREF	Somatic domain	14.3	3.4	14.5	3.0	0.6362
	Psychological domain	12.7	3.1	12.5	3.3	0.9645
	Social domain	13.3	3.6	12.5	4.1	0.2816
	Environment	13.9	2.3	14.5	2.6	0.0554
SWLS		17.4	8.4	18.2	8.1	0.5711
AIS		27.2	9.3	28.6	9.6	0.3557

Abbreviations: AIS—Acceptance of Illness Scale, SF-36—Quality of Life Assessment Questionnaire, SWLS—Satisfaction with Life Scale, WHOQOL-BREF—World Health Organization Abbreviated Quality of Life Questionnaire, $\bar{x}$—arithmetic mean, SD—standard deviation, *p*—probability value (Mann–Whitney test).

**Table 7:** Age and time since diagnosis and QoL by SF-36, WHOQOL-BREF, life satisfaction by SWLS and acceptance of infection by AIS (n = 147).

Psychometric Measures		Age (Years)	Time since Diagnosis
SF-36	Physical function	−0.24 (*p* = 0.0030 *)	0.00 (*p* = 0.9687)
	Role limitations due to physical health problems	−0.09 (*p* = 0.3056)	0.03 (*p* = 0.7499)
	Pain	−0.02 (*p* = 0.7680)	−0.01 (*p* = 0.9462)
	General health	−0.09 (*p* = 0.2584)	0.03 (*p* = 0.6793)
	Role limitations due to emotional problems	−0.09 (*p* = 0.2903)	0.15 (*p* = 0.0704)
	Vitality	−0.10 (*p* = 0.2315)	−0.08 (*p* = 0.3594)
	Social functions	−0.05 (*p* = 0.5548)	0.12 (*p* = 0.1615)
	Wellbeing	−0.03 (*p* = 0.6852)	0.01 (*p* = 0.9189)
	Physical domain	−0.17 (*p* = 0.0404 *)	0.03 (*p* = 0.7452)
	Mental domain	−0.12 (*p* = 0.1389)	0.04 (*p* = 0.6471)
	Total quality of life	−0.17 (*p* = 0.0392*)	0.01 (*p* = 0.8661)
WHOQOL-BREF	Somatic domain	−0.05 (*p* = 0.5294)	−0.05 (*p* = 0.5175)
	Psychological domain	−0.08 (*p* = 0.3571)	0.06 (*p* = 0.4363)
	Social domain	−0.12 (*p* = 0.1396)	-0.03 (*p* = 0.7224)
	Environment	−0.14 (*p* = 0.0889)	−0.07 (*p* = 0.3742)
SWLS		−0.08 (*p* = 0.3149)	−0.05 (*p* = 0.5414)
AIS		−0.14 (*p* = 0.0968)	−0.01 (*p* = 0.8834)

Abbreviations: AIS—Illness Acceptance Scale, SF-36—Quality of Life Assessment Questionnaire, SWLS—Satisfaction with Life Scale, WHOQOL-BREF—World Health Organization Abbreviated Quality of Life Questionnaire, *p*—probability value (Spearman’s rank correlation coefficient), *—statistically significant.

**Table 8:** Marital status and QoL by SF-36, WHOQOL-BREF, AIS and SWLS (n = 147).

Psychometric Measures		Marital Status						*p*
		Single (n = 71)		Married (n = 42)		Previously Married (n = 34)		
		$\bar{x}$	SD	$\bar{x}$	SD	$\bar{x}$	SD	
SF-36	Physical function	77.9	30.3	85.2	21.0	74.7	27.2	0.1859
	Role limitations due to physical health problems	59.5	40.8	77.4	40.1	39.0	44.0	0.0002 *
	Pain	63.8	30.7	72.3	25.1	60.8	35.2	0.3267
	General health	45.4	19.8	51.8	20.0	43.8	21.6	0.2730
	Role limitations due to emotional problems	64.8	44.0	85.7	33.0	62.7	47.7	0.0254 *
	Vitality	43.9	13.8	46.4	11.9	36.0	16.0	0.0061 *
	Social functions	69.9	24.8	86.9	19.5	67.6	27.7	0.0005 *
	Wellbeing	50.0	23.8	62.3	15.4	54.8	19.1	0.0089 *
	Physical domain	68.6	26.6	78.3	23.0	61.8	26.1	0.0048 *
	Mental domain	53.5	20.1	65.4	12.8	53.4	17.0	0.0014 *
	Total quality of life	61.1	21.9	71.9	16.7	57.6	19.8	0.0022 *
WHOQOL-BREF	Somatic domain	13.9	3.5	16.1	1.9	13.5	2.6	≤0.001 *
	Psychological domain	11.7	3.7	14.4	2.1	11.9	2.5	≤0.001 *
	Social domain	12.0	4.3	14.1	3.7	12.6	3.2	0.0302 *
	Environment	14.0	2.9	15.4	2.0	13.6	1.6	0.0002 *
SWLS		16.7	7.9	21.7	8.9	15.8	6.3	0.0007 *
AIS		27.6	10.3	29.5	9.3	27.8	7.8	0.4345

Abbreviations: AIS—Acceptance of Illness Scale, SF-36—Quality of Life Assessment Questionnaire, SWLS—Satisfaction with Life Scale, WHOQOL-BREF—World Health Organization Abbreviated Quality of Life Questionnaire, $\bar{x}$—arithmetic mean, SD—standard deviation, *p*—probability value (Kruskal–Wallis test), *—statistically significant.

## Data Availability

Data are available upon reasonable request.
